# Endoscopic surgery for distal femoral physeal bar resection with computed tomography-assisted navigation: a case report

**DOI:** 10.1093/jscr/rjaf972

**Published:** 2025-12-06

**Authors:** Yasutaka Masada, Tomonori Tetsunaga, Kazuki Yamada, Tomohiro Inoue, Ryuichiro Okuda, Tetsuya Yamamoto, Shin Matsumoto, Tomoko Tetsunaga, Yusuke Yokoyama, Yuki Okazaki, Toshifumi Ozaki

**Affiliations:** Department of Orthopaedic Surgery, Science of Functional Recovery and Reconstruction, Okayama University Graduate School of Medicine, Dentistry and Pharmaceutical Sciences, 2-5-1 Shikata-cho, Kitaku 700-8558, Japan; Department of Musculoskeletal Health Promotion, Faculty of Medicine, Dentistry and Pharmaceutical Sciences, Okayama University, 2-5-1 Shikata-cho, Kitaku 700-8558, Japan; Department of Medical Materials for Musculoskeletal Reconstruction, Faculty of Medicine, Dentistry and Pharmaceutical Sciences, Okayama University, 2-5-1 Shikata-cho, Kitaku 700-8558, Japan; Department of Orthopaedic Surgery, Science of Functional Recovery and Reconstruction, Okayama University Graduate School of Medicine, Dentistry and Pharmaceutical Sciences, 2-5-1 Shikata-cho, Kitaku 700-8558, Japan; Department of Orthopaedic Surgery, Science of Functional Recovery and Reconstruction, Okayama University Graduate School of Medicine, Dentistry and Pharmaceutical Sciences, 2-5-1 Shikata-cho, Kitaku 700-8558, Japan; Department of Orthopaedic Surgery, Science of Functional Recovery and Reconstruction, Okayama University Graduate School of Medicine, Dentistry and Pharmaceutical Sciences, 2-5-1 Shikata-cho, Kitaku 700-8558, Japan; Department of Orthopaedic Surgery, Science of Functional Recovery and Reconstruction, Okayama University Graduate School of Medicine, Dentistry and Pharmaceutical Sciences, 2-5-1 Shikata-cho, Kitaku 700-8558, Japan; Department of Sports Medicine, Faculty of Medicine, Dentistry and Pharmaceutical Sciences, Okayama University, 2-5-1 Shikata-cho, Kitaku 700-8558, Japan; Department of Advanced Rehabilitation Medicine for the Musculoskeletal System, Okayama University, 2-5-1 Shikata-cho, Kitaku 700-8558, Japan; Center for Education in Medicine and Health Sciences, Okayama University, 2-5-1 Shikata-cho, Kitaku 700-8558, Japan; Department of Orthopaedic Surgery, Faculty of Medicine, Dentistry and Pharmaceutical Sciences, Okayama University, 2-5-1 Shikata-cho, Kitaku 700-8558, Japan

**Keywords:** physeal bar, computed tomography, navigation

## Abstract

The formation of physeal bars, or bony bridges, following growth plate injuries can cause complications such as angular deformities or discrepancies in leg length. The management strategies for these depend on factors such as the bar’s location, extent, and residual growth potential. Herein, we describe the case of a 14-year-old male with a valgus knee deformity caused by a distal femoral physeal bar. The patient underwent endoscopic resection of the bar, assisted by computed tomography-based navigation and intraoperative O-arm imaging. This minimally invasive technique facilitated safe and accurate removal of the lesion with less risk of complications such as cortical perforation or injury to adjacent neurovascular structures compared to traditional approaches. The patient experienced favorable postoperative outcomes, including restored knee range of motion and full symptom resolution. This approach demonstrates the clinical value of integrating endoscopy with advanced navigation systems during the surgical treatment of physeal bars.

## Introduction

Physeal bars, resulting from epiphyseal plate injury, disrupts growth in children [1]. The most frequent etiology is trauma, along with infections, neoplasms, burns, coagulation disorders, or idiopathic factors [2]. Clinical manifestations vary by location: peripheral bars cause angular deformities, whereas central ones cause limb length discrepancies and alignment abnormalities [3–5].

Management depends on growth potential and bar characteristics. Non-operative strategies may suffice with minimal anticipated growth or limited impact of the bar on alignment and length [3]. Surgical intervention is considered when deformity or limb length discrepancy correction is needed [6, 7]. Physeal bar resection aims to restore growth and correct alignment, although safe access and complete removal are challenging and risks iatrogenic damage [8].

Endoscopic techniques are used to manage chronic osteomyelitis involving the physis, offering minimal surgical trauma [9]. Compared to open methods, endoscopy improves visualization and reduce invasiveness [1].

Computed tomography (CT)-based navigation systems in orthopedic surgery are increasingly adopted, demonstrating favorable results for various applications [10]. The Stealth Station™ system (Medtronic, Dublin, Ireland) automates registration of surgical instruments (pointers, taps, and drills) [11]. Integrated with the O-arm™ (Medtronic, Dublin, Ireland), a mobile intraoperative CT imaging platform, surgeons can gain real-time three-dimensional guidance [12]. Though increasingly used in spinal and pelvic procedures; its application in physeal bar resection is novel [12]. We report a case of physeal bar resection using a combined endoscopic and CT-navigated approach. Informed consent was obtained from both the patient and his legal guardians for the publication of this report

## Case report

A 14-year-old boy presented with progressive right knee valgus deformity identified 10 months earlier, with no relevant medical or trauma history. His height and weight were 169.8 cm and 54.2 kg, respectively.

**Figure 1:**
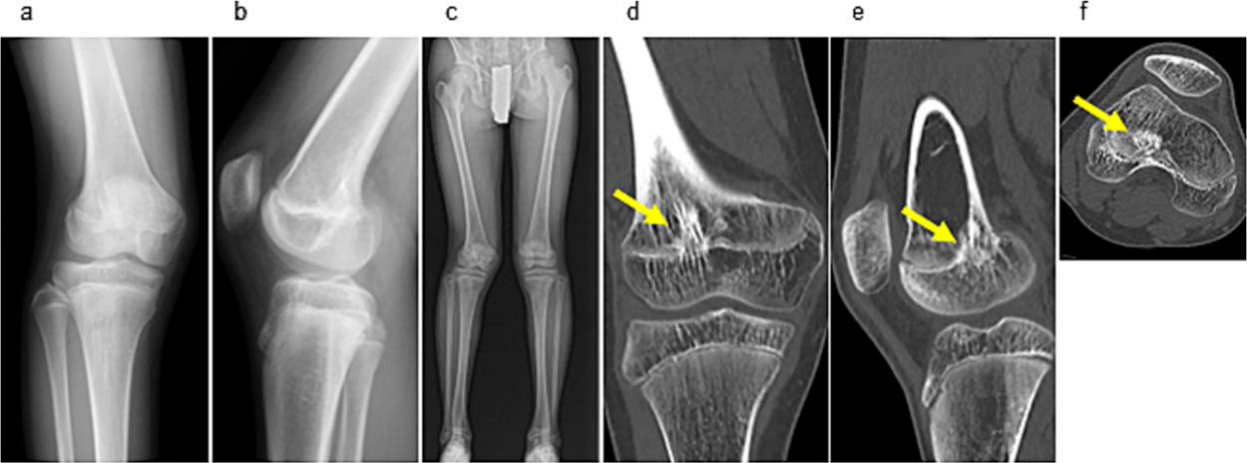
Preoperative radiographic and CT findings. (a) Anteroposterior radiograph of the right knee. (b) Lateral radiograph of the right knee. (c) Full-length standing anteroposterior radiograph of the lower extremities showing valgus deformity (femorotibial angle: right, 165°; left, 172°). (d) Coronal CT image. (e) Sagittal CT image. (f) Axial CT image showing early epiphyseal closure of the distal femur caused by a physeal bar (arrow), located adjacent to the posterior cortical bone.

Examination revealed full range of motion in the affected knee (0–140°) and no pain during walking. Spinal malleolar distance assessment showed a leg length discrepancy of 30 mm (shorter right limb). Radiography showed valgus alignment with a femorotibial angle (FTA) of 162° on the right leg and 172° on the left (Fig. 1a–c). CT revealed early epiphyseal closure of the distal femur, indicating physeal bar formation near the posterior cortical bone (Fig. 1d–f). Accordingly, physeal bar resection was planned.

**Figure 2:**
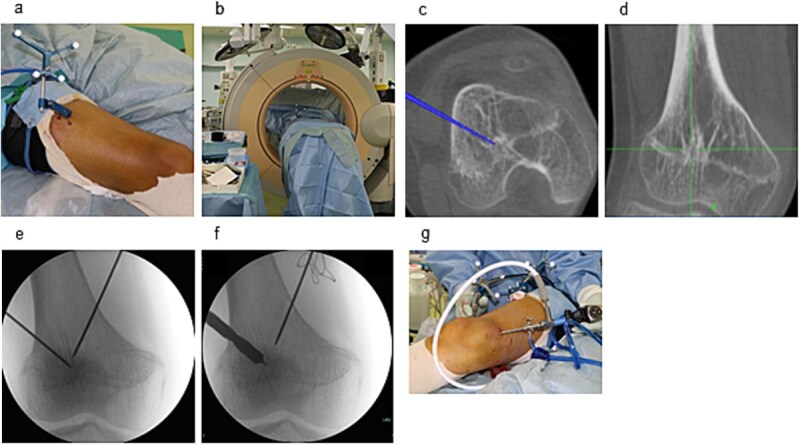
Intraoperative setup and portal creation. (a) Two 3-mm pins were inserted into the femoral diaphysis to secure the tracker of the Stealth Station™ system. (b) Intraoperative CT was performed using the O-arm™ system and integrated with the navigation system. (c) The location and trajectory to the physeal bar were identified using navigation. (d) Two 1.6-mm Kirschner wires were inserted from the medial and lateral aspects toward the physeal bar under fluoroscopic guidance. (e) Drill portals (8 mm) were created along the wire tracts. (f) A 4-mm arthroscope was inserted through one portal for endoscopic visualization.

Surgery was performed under general anesthesia with the patient supine. Two 3 mm reference pins were inserted into the femoral diaphysis to secure the Stealth Station™ navigation tracker (Fig. 2a). Intraoperative CT using the O-arm™ system integrated with the navigation platform was used to identify the physeal bar’s location and orientation (Fig. 2b–d).

**Figure 3:**
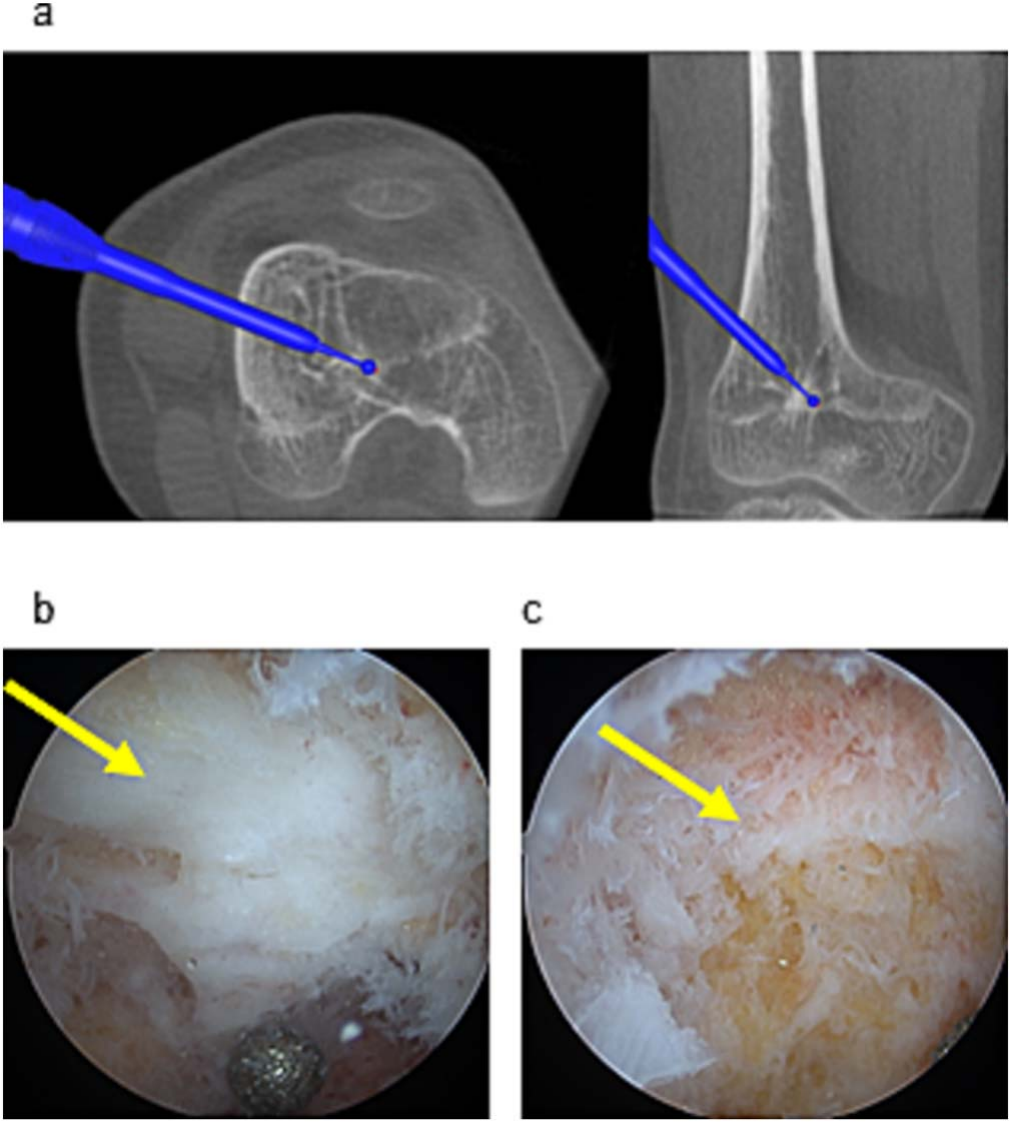
Endoscopic and navigational views during resection. (a) Physeal bar resection was performed using a high-speed drill under navigation guidance. (b) The physeal bar (arrow) was directly visualized endoscopically. (c) Following excision, residual growth cartilage (arrow) was observed at the site.

**Figure 4:**
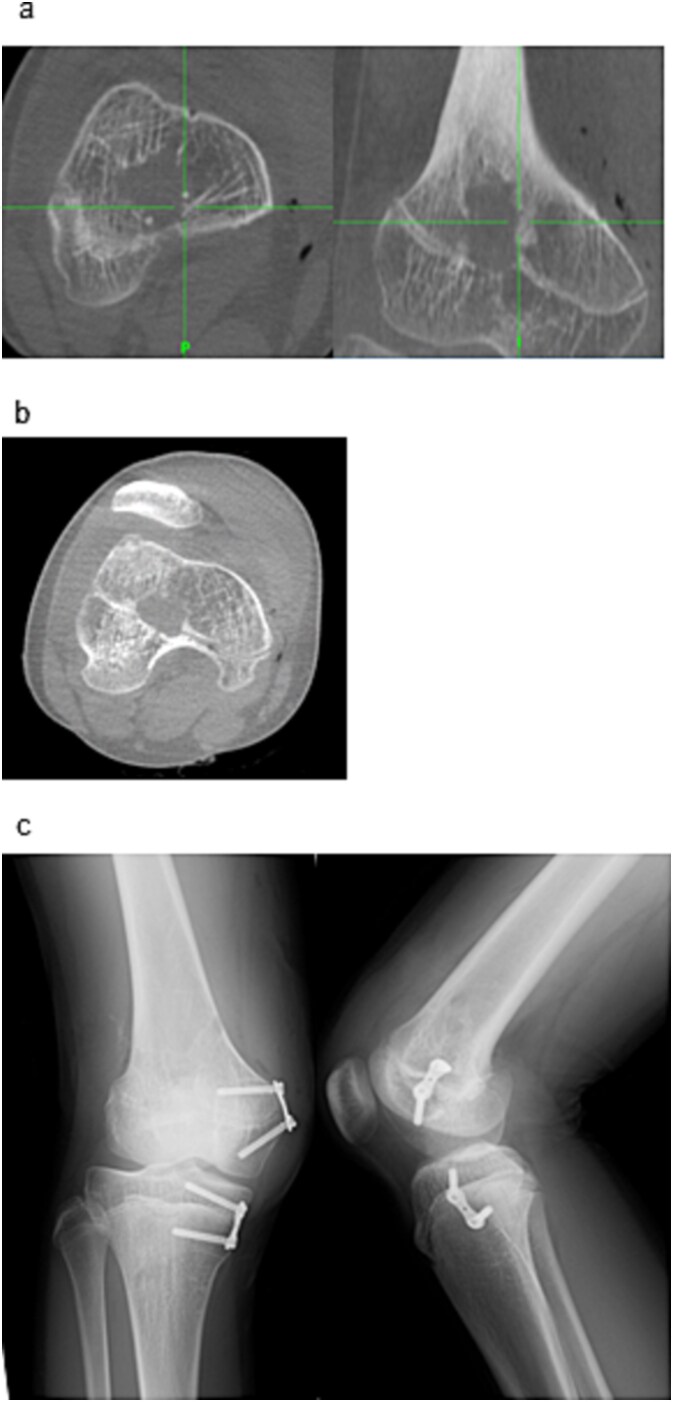
Post-resection imaging and implant placement. (a) Post-resection CT confirming complete removal of the physeal bar. (b) No evidence of cortical perforation was seen. (c) Postoperative radiographs (anteroposterior and lateral) showing placement of the eight-plate across the distal medial femur and proximal medial tibia.

Two 1.6 mm Kirschner wires were inserted percutaneously from the medial and lateral distal femoral sides toward the bar (Fig. 2e), and 8 mm drill portals were created along the wires (Fig. 2f). A 4 mm arthroscope (Stryker Corporation, Kalamazoo, MI, USA) was introduced through one portal for direct visualization of the physeal bar (Fig. 2g). A Stealth-Midas™ high-speed drill (Medtronic) was advanced through the contralateral portal. Resection was performed under concurrent navigational and endoscopic monitoring (Fig. 3a and b). Complete bar removal was confirmed visually and on the navigational display (Fig. 3c). Intraoperative CT via O-arm confirmed a thorough excision (Fig. 4a) with no cortical perforation (Fig. 4b).

**Figure 5:**
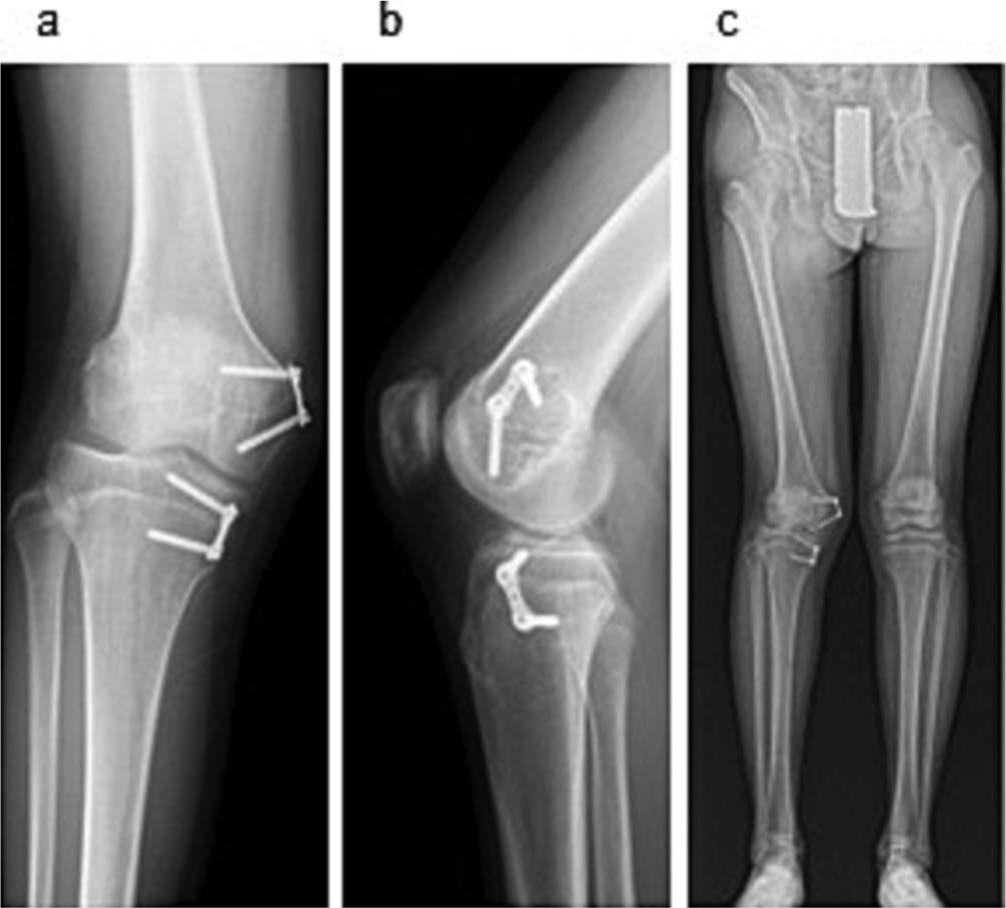
Radiographic findings at 24-month follow-up. (a) Anteroposterior radiograph of the right knee. (b) Lateral radiograph of the right knee. (c) Full-length standing anteroposterior radiograph of the lower extremities.

To correct the valgus deformity, an eight-plate (MC Medical Corporation, Tokyo, Japan) was inserted, across the distal medial femur and proximal medial tibia (Fig. 4c). Mild thigh swelling from the irrigation fluid occurred postoperatively; with no signs of compartment syndrome or wound complications. Weight-bearing began the next day with crutch-assisted ambulation guided by pain tolerance. He was discharged on postoperative Day 6, ambulating independently with crutches. At the 24-month follow-up, he reported no pain and improved right knee range of motion (0–140°) (Fig. 5a–c). Radiography showed that the FTA had improved to 168°.

## Discussion

Conventional physeal bar resection methods via open surgery [1] or fluoroscopic guidance [2] pose challenges in achieving complete excision without damaging surrounding tissues. Incomplete resection leads to recurrence or necessitates revision procedures.

Endoscopic approaches address these limitations, improving visualization and minimizing invasiveness [9, 13]. Approximately 70% of patients who undergo endoscopically-assisted physeal bar resection experience favorable longitudinal growth outcomes [13]. However, these procedures often continue to depend on fluoroscopy, which has limited capabilities in accurate three-dimensional assessment of structures and trajectories.

Integrating endoscopic visualization with CT-based navigation enhances surgical precision for physeal bar resection [14]. Navigational systems accurately localizes lesions and guide drilling trajectories; endoscopy facilitates real-time visualization of the resection zone. It is particularly effective for centrally located bars or those adjacent to critical anatomical structures. In this case, the physeal bar was situated near the posterior cortex of the distal femur, close to the neurovascular bundle, posing a substantial risk of cortical perforation and soft tissue injury. To mitigate these risks, intraoperative navigation provided accurate three-dimensional tracking of the drill tip, whereas endoscopy allowed for direct assessment of the resection area. Intraoperative CT confirmed complete bar removal and prevented under-resection.

In this case, the etiology was idiopathic, as no definitive cause of physeal damage was identified (no evidence of trauma, infection, or coagulopathy). Despite limited growth potential, the patient and his family preferred earlier intervention over corrective osteotomy after skeletal maturity. Minimally invasive resection was therefore performed. Clinical outcomes were favorable despite the short follow-up and reduced growth potential.

A limitation of this technique is radiation exposure. Although radiation dose was not quantified, pediatric studies suggest O-arm radiation is ~80% that of conventional CT and unrelated to body size, even with repeated scans [15]. Only one intraoperative scan was used in our protocol, minimizing radiation burden.

Endoscopic visualization combined with CT-based navigation for physeal bar resection proves to be safe, precise, minimally invasive, and useful in anatomically complex cases. Further studies with larger cohorts and longer follow-up are warranted to validate long-term efficacy and safety, particularly regarding growth potential and cumulative radiation exposure in pediatric patients.
